# Using personalized prognosis in the treatment of relapsing multiple sclerosis: A practical guide

**DOI:** 10.3389/fimmu.2022.991291

**Published:** 2022-09-27

**Authors:** Bart Van Wijmeersch, Hans-Peter Hartung, Patrick Vermersch, Maura Pugliatti, Carlo Pozzilli, Nikolaos Grigoriadis, Mona Alkhawajah, Laura Airas, Ralf Linker, Celia Oreja-Guevara

**Affiliations:** ^1^ Universitair Multiple Sclerosis (MS) Centrum, Hasselt-Pelt, Belgium; ^2^ Noorderhart, Revalidatie & Multiple Sclerosis (MS), Pelt, Belgium; ^3^ REVAL & BIOMED, Hasselt University, Hasselt, Belgium; ^4^ Department of Neurology, Medical Faculty, Heinrich-Heine-University Düsseldorf, Düsseldorf, Germany; ^5^ Brain and Mind Center, University of Sydney, Sydney, NSW, Australia; ^6^ Department of Neurology, Palacky University Olomouc, Olomouc, Czechia; ^7^ University Lille, Inserm U1172 LilNCog, Centre Hospitalier Universitaire (CHU) Lille, Fédératif Hospitalo-Universitaire (FHU) Precise, Lille, France; ^8^ Department of Neuroscience and Rehabilitation, University of Ferrara, Ferrara, Italy; ^9^ Unit of Clinical Neurology, San Anna University Hospital, Ferrara, Italy; ^10^ Department of Human Neuroscience, Sapienza University, Rome, Italy; ^11^ B’ Department of Neurology, Aristotle University of Thessaloniki, Thessaloniki, Greece; ^12^ Neuroscience Center, King Faisal Specialist Hospital and Research Center, Riyadh, Saudi Arabia; ^13^ Turku University Hospital and University of Turku, Turku, Finland; ^14^ Department of Neurology, University Hospital Regensburg, Regensburg, Germany; ^15^ Department of Neurology, Hospital Clínico San Carlos, Instituto de Investigación Sanitaria del Hospital Cliínico San Carlos (IDISSC), Madrid, Spain; ^16^ Department of Medicine, Faculty of Medicine, Universidad Complutense de Madrid, Madrid, Spain

**Keywords:** multiple sclerosis, prognosis, clinical parameters, magnetic resonance imaging (MRI), biomarkers, treatment, optical coherence tomography, evoked potentials

## Abstract

The clinical course of multiple sclerosis (MS) is highly variable among patients, thus creating important challenges for the neurologist to appropriately treat and monitor patient progress. Despite some patients having apparently similar symptom severity at MS disease onset, their prognoses may differ greatly. To this end, we believe that a proactive disposition on the part of the neurologist to identify prognostic “red flags” early in the disease course can lead to much better long-term outcomes for the patient in terms of reduced disability and improved quality of life. Here, we present a prognosis tool in the form of a checklist of clinical, imaging and biomarker parameters which, based on consensus in the literature and on our own clinical experiences, we have established to be associated with poorer or improved clinical outcomes. The neurologist is encouraged to use this tool to identify the presence or absence of specific variables in individual patients at disease onset and thereby implement sufficiently effective treatment strategies that appropriately address the likely prognosis for each patient.

## Introduction

Multiple sclerosis is a highly heterogeneous disease of the central nervous system (CNS) that affects over 2.8 million people worldwide (1). The disease course can vary markedly, with overt clinical and imaging activity (relapses, fatigue, cognitive impairment, brain/spinal cord lesions, etc.) seen in some people with MS compared to a relatively benign course in others. While MS neurologists have at their disposal a growing list of disease-modifying therapies (DMTs) with different modes of action, efficacies, routes of administration, and concomitant safety characteristics, treatment of the MS patient does not follow a one-size-fits-all approach.

When seeing a person with relapsing-remitting MS (RRMS) for the first time, the MS neurologist must address a fundamental question: “What is the nature of the disease activity and likely prognosis of the person with MS before me?” As the course of action taken by the neurologist at this point will likely prove pivotal to the long-term outcomes of this patient, the challenge here is for correct decisions to be taken concerning the DMT to be used and the treatment regimen (escalation versus induction) to be followed so that the best possible result for the patient is achieved. Local prescribing guidelines usually reserve higher efficacy DMTs for patients who have failed first-line treatments or who have ‘high disease activity’ as indicated by clinical (relapses) or imaging [magnetic resonance imaging (MRI) lesion activity] features. However, we propose that a much more proactive approach on the part of the neurologist is needed if patients’ long-term needs are to be fully addressed from the outset and if a level of care is to be provided that is over and above that of simply administering DMTs in order of efficacy as per local guidelines. Herein lies the difficulty for the busy neurologist, who might see hundreds of patients every month, to be fully cognizant of each patient’s treatment needs.

We propose that the correct approach to treating persons with MS is according to each person’s prognosis, and not solely on the basis of ‘clinical or imaging features’ at the initial MS diagnosis. A patient with a poor prognosis may not necessarily have ‘highly active disease’ but may exhibit numerous clinical disease characteristics that have been shown to be associated with poor long-term outcomes and indicating the need for a more effective treatment strategy to be implemented. Excellent reviews have been published outlining the scientific basis for the prognostic value of many clinical, imaging, biomarker, and related parameters in MS patients [see e.g (2).]. In the present paper, we attempt to integrate this information into a compact ‘prognosis tool,’ drawing as well on our own years of experience treating MS patients. Our objective is to provide neurologists with a practical ‘checklist’ guide to establishing the likely prognosis of patients based primarily on baseline clinical parameters that can also be reassessed at periodic follow-up visits. Though not an all-encompassing, scientifically validated tool, most of the items in the checklist can be assessed in a hospital/clinical setting and do not require complex imaging or advanced analytical techniques. Importantly, the guide will allow neurologists to identify ‘red flag’ parameters in the MS patient profile that are related to poorer long-term prognosis. The presence in a patient’s profile of any parameters indicating poor prognosis should be a warning sign to the neurologist that close attention needs to be paid to this patient and that treatment strategies – escalation from first- to second- and later-lines versus the immediate use of high efficacy induction therapies – require careful consideration and implementation in a timely manner. It must be emphasized that the decision to treat with high-efficacy DMTs should be based on patients with poor prognosis and on patients with active disease.

## Methodology

The concepts and recommendations presented here by the authors were developed over the course of several meetings held by a panel of experts belonging to the ParadigMS Foundation, a private, non-profit entity whose primary endeavour is to provide educational materials to the MS medical community. Consecutive iterations of the prognosis tool were reviewed and revised until the present version was arrived at.

This practical guideline was developed by first considering objectives from the points of view of the neurologist and the patient, and then defining the most relevant and easily measurable parameters that impact on and signify prognosis.

## Structure of the prognosis tool

### Section I: The setting of objectives: Finding common ground between patients’ needs and neurologists’ treatment goals

The assumption here is that the patient sitting before the neurologist has a confirmed diagnosis of MS according to the Lublin classification (3). From this point onwards, every patient will likely have a different disease course and therefore a different prognosis. Based on the initial features of the disease, it is important for both the patient and the neurologist to clarify their expectations of the treatment approach to be followed. In this way, the treatment decision taken would need to reflect a common understanding of the objectives held by patient and neurologist; it is highly likely that these objectives will be different if the clinical reality held by the neurologist and the potentially idealistic notions of the patient are compared. For example, a certain DMT may provide symptom relief and improved quality of life, which would be highly desirable outcomes for the patient, and yet the presence of brain lesion activity on MRI scans may require a stronger DMT in the opinion of the neurologist, but one that has more pronounced side effects. For this reason, the MS patient and neurologist need to reach common ground concerning objectives that are acceptable to both.

Aside from the patient’s perspective, we believe that an appropriate starting objective for the neurologist prior to treatment initiation is NEDA (No Evidence of Disease Activity)-3 at 2 years. This is defined as no relapse activity, no new MRI lesions, and no disability progression after two years of treatment. Rotstein et al. (2015) (4) showed that NEDA-3 at 2 years had a positive predictive value of 78.3% for no progression (change in Expanded Disability Status Scale (EDSS) score ≤0.5) at 7 years. If the patient’s prognosis prior to treatment initiation on a certain DMT makes this objective seem unrealistic, then consideration should be given to starting the patient on a stronger therapy. Each follow-up visit made by the patient can be seen as an opportunity to reassess the patient’s disease status and determine if the treatment is working satisfactorily.

### Section II: Defining parameters that impact on prognosis. Which ‘red flags’ must be paid attention to, and what parameters are included in the prognosis tool?

The achievement of objectives and justification of the treatment decision are highly dependent on the patient’s initial prognosis. It would be futile to start a patient on a low-efficacy DMT if the initial clinical profile suggests a poor prognosis. To this end, the patient’s initial prognosis needs to be well established.

Our prognosis tool consists of parameters categorised into five key areas: Demographic, Clinical, MRI, Biomarkers, and Evoked Potentials (EPs) /Optical Coherence Tomography (OCT). Some of these parameters have binary outcomes (two possible options to choose from), whereas others have ranges over which the prognostic weight can be distributed over several values.

#### Demographic factors impacting on prognosis

Responses to this section in the prognosis tool can be viewed in **Table 1** (Section A). A PDF version of the table can be downloaded from the following website: https://paradigms.foundation/prognosis/

**Table 1:** Practical checklist for using personalized prognosis in MS.

(A) Demographic factors impacting on prognosis	Item	Better prognosis 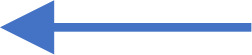		Poorer prognosis 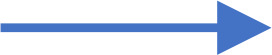		
	***Age (years)***	**<30**	**<40**	**≥40**	**≥50**	
	Older age has been associated with poorer prognosis in MS	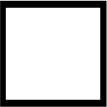	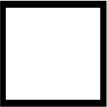	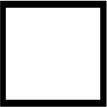	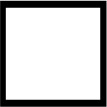	
	***Sex***	**Female**		**Male**		
	Studies show that disability worsening milestones are met earlier in males	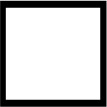		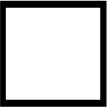		
	***Serum Vitamin D Levels***	**25(OH)D level**		**25(OH)D level**	**25(OH)D level**	
		**>75 nmol/L**		**50-75 nmol/L**	**<50 nmol/L**	
	Vitamin D is a steroid hormone that is involved in many important physiological functions in humans and has been strongly implicated in MS disease activity and disability progression. A negative correlation exists between 25(OH)D levels and disability across all forms of MS (RRMS, SPMS, PPMS)	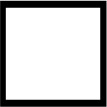		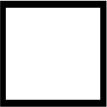	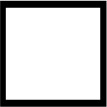	
	***Smoking***	**Non-smoker**		**Smoker**		
	Smoking of tobacco products has long been associated with risk of MS and of disease progression	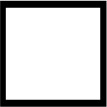		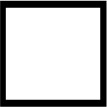		
	***Comorbidities***	**No Comorbidities**		**Comorbidities**		
				**1**	**2**	**≥3**
	Conditions such as cardiovascular diseases, obesity and psychiatric disorders (depression, anxiety) have been associated with disability progression and reaching disability milestones earlier	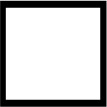		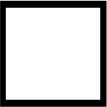	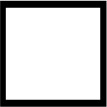	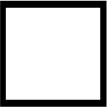
(B) Clinical manifestations impacting on prognosis						
** **	***Disease subtype***	**Relapsing forms of MS**		**Progressive forms of MS**		
** **	Disability progression is indicative of a poorer prognosis given the paucity of effective treatment options for progressive versus relapsing forms of MS	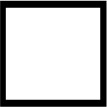		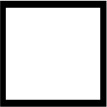		
** **	***Relapse rate***	**≤1 in 2 years since diagnosis**	**<1 per year**	**1 in previous year**	**≥2 per year**	
** **	The relapse frequency in the first two years since diagnosis is associated with more rapid progress to disability milestones	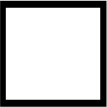	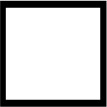	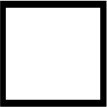	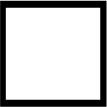	
** **	***Interval between relapses***	**≥2 years**	**>1 year**	**<1 year**	**<6 months**	
** **	The time between first and second relapses is associated with more rapid progress to disability milestones	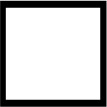	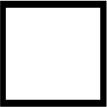	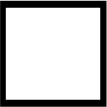	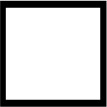	
** **	***Recovery from relapse***	**Full recovery**		**Partial recovery**		
** **	Complete recovery is a positive prognostic indicator that predicts a slower progression to irreversible disability landmarks	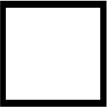		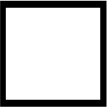		
** **	***EDSS at diagnosis***	**EDSS score ≤2**		**EDSS score >2**		
** **	** **Higher EDSS values at diagnosis are associated with a poorer prognosis	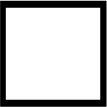		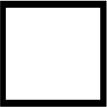		
** **	***Brainstem, cerebellar or spinal cord onset***	**Absent**		**Present**		
** **	Symptomatic involvement of the pyramidal, cerebellar, sphincteric or visual systems at MS disease onset is unfavourable	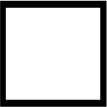		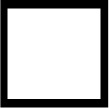		
** **	***Form of symptomatic onset***	**Monosymptomatic**		**Polysymptomatic**		
** **	A polysymptomatic onset of MS activity has been associated with poorer recovery and greater disability progression with time	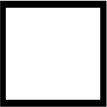		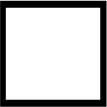		
** **	***Cognitive deficits*** Cognitive impairment at baseline (deficits in information processing speed and verbal memory) are correlated with higher EDSS scores 5 to 7 years later	**No cognitive decline** 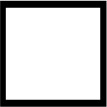		**Mild cognitive decline** 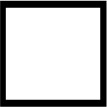	**Moderate / Severe cognitive decline** 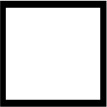	
(C) MRI observations impacting on prognosis						
	***Number of T2 lesions***	**Low number of T2 lesions**		**High number of T2 lesions**		
		**1-4**		**5-9 ≥10**		
	Numerous studies point to the fact that the number and volume of brain T2 lesions on MRI scan at diagnosis is correlated to long-term disability outcomes	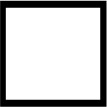		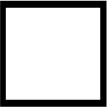 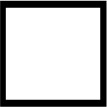		
	***Gadolinium(Gd)-enhancing, infratentorial, and spinal cord lesions***					
		**No Gd-enhancing lesions**		**Presence of Gd-enhancing lesions**		
		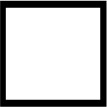		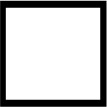		
	The presence of these lesions in relapsing MS patients at diagnosis or early disease (1-3 years) is correlated with poor long-term outcomes such as conversion to SPMS or increased disability as measured by EDSS	**No infratentorial lesions**		**Presence of infratentorial lesions**		
		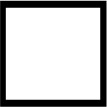		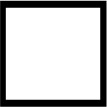		
		**No spinal cord lesions** 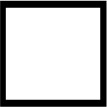		**Presence of spinal cord lesions** 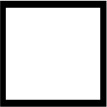		
		
	***T1 Black holes***	**No T1 black holes**		**Presence of T1 black holes**		
	T1 hypointense (black holes) lesions have been associated with demyelination, axonal loss, and neurodegeneration	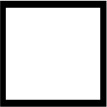		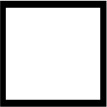		
(D) Biomarkers						
** **	***Oligoclonal bands (OCBs)***	**No OCBs**	**Presence of OCBs**		**Presence of IgM OCBs**	
** **	The presence of OCBs is associated with poorer prognosis, whereas the absence of OCBs is associated with to a more benign disease course	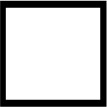	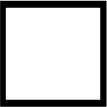		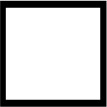	
** **	***Neurofilament Light (NfL) chain levels in serum***	**NfL levels <80th percentile for healthy controls**	**NfL levels ≥80th percentile for healthy controls**			
** **	Evidence from numerous studies involving MS patients points to a correlation of high NfL levels with disability progression.					
** **		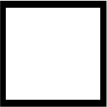	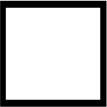			
(E) Optical Coherence Tomography (OCT) and Evoked Potentials (EPs)
** **	***Retinal nerve fibre layer (RNFL) properties***	**RNFL thickness >88 μm at baseline**	**RNFL thickness ≤88 μm at baseline**			
** **	Baseline RNFL thinning is indicative of subclinical axonal damage and early neurodegeneration that can be measured by OCT	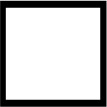	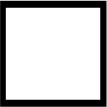			
** **	***Evoked potential characteristics***	**0 or 1 abnormal EP**	**2 abnormal EPs**		**3 or more abnormal EPs**	
** **	Abnormal somatosensory, motor, or global EP scores at baseline are correlated with later disability progression	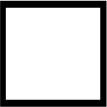	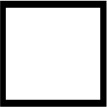		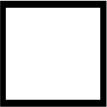	

##### Age

Older age has been associated with poorer prognosis in MS. For example, older individuals at onset had more rapid disability worsening (5–8), were at greater risk of converting to secondary progressive MS (SPMS) (8) and had a higher likelihood of incomplete or poorer recovery following relapse activity (9, 10). This is probably because older patients are likely to have had subclinical disease activity for a longer time, resulting in reduced ‘brain reserve’ or, in other words, a reduced capacity to compensate for neurodegenerative damage. In a population-based cohort study (6), the time for progression from MS diagnosis to SPMS was significantly reduced in patients with late onset MS disease (defined as ≥50 years). For these reasons, we consider older age to be associated with a poorer prognosis.

Scoring: Age brackets above and below 40 years indicating progressively poorer and better prognosis, respectively.

##### Sex

While the number of females affected by MS is proportionally much greater than that of males (3:1) (11), studies show that disability worsening milestones are met earlier in males (2, 12), and that MRI brain lesions (13) and cognitive impairment (14, 15) tend to be more severe.

For these reasons, we include the male gender as a risk factor for poorer prognosis.

Scoring: Binary outcome – Male/female.

##### Vitamin D levels

Vitamin D is a steroid hormone that is involved in many important physiological functions in humans and has been strongly implicated in MS disease activity and disability progression (2, 11, 16). Low Vitamin D levels (and of its metabolite 25(OH)D) early in the RRMS disease course have been associated higher relapse rates (17), higher MRI lesion activity, and an increase in the annualised change in EDSS (18). A meta-analysis of 14 studies showed primary progressive MS (PPMS) a significant negative correlation between 25(OH)D levels and disability across all forms of MS (RRMS, SPMS, PPMS) (19). As noted by Smolders et al. (2019) (20), the presence of low 25(OH)D levels early in the disease is indicative of a higher indicative of patients with a high risk of an active inflammatory disease course. Taken together, these findings suggest that Vitamin D levels could serve as an important biomarker for disease activity and should be assessed in the prognostic workup. Concerning the manner by which vitamin D levels could be incorporated into the prognosis tool, the BENEFIT trial (18) highlighted that serum 25(OH)D levels in the first 12 months in patients with clinically isolated syndrome (CIS) were predictive of disability outcomes at 5 years. In that study and others (18, 21, 22), a serum 25(OH)D concentration less than 50 nmol/L was considered to represent hypovitaminosis D, while 50-75 nmol/L was suggestive of Vitamin D insufficiency, and concentrations greater than 75 nmol/L indicative of normal levels.

Scoring: We suggest that a serum 25(OH)D concentration of <50 nmol/L may indicate a poorer prognostic outcome, while >50 nmol/L is preferred.

##### Smoking

The smoking of tobacco products has long been associated with an increased risk of MS and of disease progression. Smoking induces a proinflammatory environment that is linked to numerous manifestations of exacerbated outcomes in MS patients. These include greater brain lesion loads on MRI scans (23–27), as well as increased rates of clinical relapse (26, 28, 29), brain atrophy (24, 27, 30) and disability progression (16, 31, 32). Moreover, a poorer response to DMTs (26, 28) and a greater risk of associated comorbidities have been described in MS patients who smoke (26). While prognostic outcomes are considered to be negatively affected in people who smoke, this is a modifiable risk factor given that measures can be taken to reduce or stop the habit (26).

Scoring: Binary response, with ‘Smoker’ indicating a poorer prognosis versus ‘Non-smoker.’

##### Comorbidities

Conditions such as cardiovascular diseases (33), obesity (11, 16, 34, 35) and psychiatric disorders (depression, anxiety) have been associated with disability progression and reaching EDSS markers earlier (2). For the purposes of the prognosis tool, we are specifically interested here in comorbidities that may impact on MS disease course progression. As outlined by Magyari & Sorensen (2020) (36), several studies have addressed this point. For example, diabetes mellitus, hypertension, and chronic obstructive pulmonary disease impact 3-year outcomes related to walking speed, self-reported disability, and depression (37). On the other hand, in a Canadian study, migraine and hyperlipidemia were specifically associated with an increase in relapse activity over 2 years, as was the presence of three or more of any of the following: migraine, hypertension, diabetes mellitus, heart disease, hyperlipidemia, depression or anxiety (38). Other comorbidities associated with enhanced relapse activity or disability progression were vascular comorbidities, rheumatoid arthritis, anaemia, and autoimmune comorbidities (psoriasis, thyroid disease, and type 2 diabetes mellitus) (36). Comorbidities are included in the prognosis tool given their association, when present, with poorer long-term outcomes.

Scoring: A good prognosis is indicated by the absence of comorbidities. A poorer prognosis will result from the presence of 1, 2, ≥3 concomitant comorbidities of increasing weight contribution.

#### Clinical manifestations impacting on prognosis

Responses to this section in the prognosis tool can be viewed in **Table 1** (Section B).

##### Disease subtype

As discussed above, most DMTs target inflammatory activity in MS, which is typically associated with relapsing forms of the disease and is therefore treatable to some extent. In contrast, a patient with PPMS at the initial consultation, or an RRMS patient who shows evidence of transition to SPMS on follow-up visits, is accruing irreversible disability, and at a faster rate (12, 39). Such progression is indicative of a poorer prognosis given the paucity of effective treatment options for smouldering disease or progressive forms of MS (40–42).

Scoring: Binary outcome – Relapsing (better prognosis)/Progressive (poorer prognosis).

##### Relapse rate, interval between first and second relapse, and recovery from first relapse

Scalfari et al. (2010) (43) showed that the relapse frequency in the first two years since diagnosis, along with the time between first and second relapses were both associated with more rapid progress to disability milestones. These two parameters are highly informative and easily measured, and thus included in the prognosis tool. A further relapse-related parameter is the level of recovery from a first relapse. Complete recovery is a positive prognostic indicator that predicts a slower progression to irreversible disability landmarks (44) and the presence of neurological reserve to compensate for damage (42, 45). In contrast, incomplete recovery is associated with faster disability progression (2, 44, 46, 47).

Scoring:

Relapse rate:

Better prognosis: Weighted values of ≤1 in two years since diagnosis and <1 per year
Poorer prognosis: 1 in previous year and ≥2 per year
***Interval between relapses:***
Better prognosis: weighted values of ≥2 years and >1 year
Poorer prognosis: weighted values of <1 year and <6 months
***Recovery:***
Better prognosis: fully recovery
Poorer prognosis: incomplete recovery

##### EDSS at diagnosis

In a recent, systematic review of prognosis prediction models for RRMS based on a sample of 30 studies, Brown et al. (2020) (48) showed that the single most frequently included predictor in prognostic models was baseline EDSS. In a study by Rudick et al. (2010) (49), it was shown that patients with a baseline EDSS score ≤2.0 were significantly less likely to progress to EDSS scores of 4.0, 5.0, 6.0, and 7.0 over an 8-year follow-up than those with a baseline EDSS score of >2.0. This fits with our general experience that higher EDSS values at diagnosis are associated with a poorer prognosis, thus supporting the inclusion of baseline EDSS as a component of the prognosis tool.

Scoring: EDSS score ≤2.0 (better prognosis); EDSS score >2.0 (poorer diagnosis).

##### Brainstem, cerebellar or spinal cord onset

A 2001 study by Amato and Ponziani (50) highlighted that symptomatic involvement of the pyramidal, cerebellar, sphincteric or visual systems at MS disease onset influences the long-term EDSS progression and is indicative of an unfavorable prognosis. In our prognosis tool, as clinically isolated syndrome patients with optic neuritis had a lower risk of disability progression (51), we have left ‘visual systems’ out of the list of parameters. In contrast, numerous studies have shown that ‘long tract’ signs (pyramidal, cerebellar) result in poorer outcomes, with these signs mostly influenced by infratentorial and spinal cord lesions (52–57).

Scoring: Binary (Yes/No) response to note the presence or absence of symptom onset involving the brainstem, cerebellar or spinal cord systems at MS diagnosis.

##### Form of symptomatic onset (monosymptomatic/polysymptomatic)

A polysymptomatic onset of MS activity has been associated with poorer recovery and greater disability progression with time (58–61). We consider this to be sufficiently well-described in the literature, is seen in our own clinical practice, and as it is relatively easily assessed it would be a useful parameter for inclusion in the prognosis tool.

Scoring: Binary outcome – Monosymptomatic/Polysymptomatic.

##### Cognitive deficit

It is well-recognised by patients, neurologists, and caregivers alike that cognitive decline in MS can be highly debilitating given its impact on social interactions, employment, and quality of life. Deloire et al. (2010) (62) showed that in a cohort of 45 MS patients, cognitive impairment at baseline (deficits in information processing speed and verbal memory) correlated with higher EDSS scores 5 and 7 years later. Cognitive impairment tests such as the Brief International Cognitive Assessment for Multiple Sclerosis (BICAMS), the Symbol Digit Modalities Test (SDMT), which assesses information processing speed, and the Selective Reminding Test (SRT), which tests verbal memory, are highly informative of disability worsening and may serve as an important component of the proposed prognosis tool. According to Oset et al. (2020) (63), the SDMT appears to be the most rapid test to perform and provides a highly informative means for assessing cognitive impairment early in MS. Moreover, a clinically meaningful change of 8 points in the SDMT has been reported, which would allow cognitive decline in follow-up visits to be assessed and quantified (64). For tests such as the BICAMS, values of 1.5 or 2 standard deviations below the control mean or below the 5^th^ percentile of the control group are considered indicative of cognitive impairment (63).

Scoring: Better prognosis: No cognitive decline.

Poorer prognosis: 1. Mild cognitive decline; 2. Moderate/severe cognitive decline.

#### MRI observations impacting on prognosis

Responses to this section in the prognosis tool can be viewed in **Table 1** (Section C).

The use of MRI to measure brain lesion activity is a commonly available diagnostic technique forming part of routine clinical practice at most major medical centres. Significant advances in instrument characteristics and protocols over the last 25 years has pushed MRI and other imaging modalities to the forefront of MS diagnosis, follow-up, and research.

##### Number of T2 lesions

Numerous studies point to the fact that the number and volume of brain T2 lesions on MRI scan at diagnosis, and their change early in the disease course, are correlated to long-term disability outcomes (65–73). Given that MRI forms part of the diagnostic workup for MS in most centres, the T2 lesion number at baseline should be a readily measurable parameter providing an important indication of likely prognosis.

Scoring: Better prognosis: ≤4 T2 lesions.

Poorer prognosis: (weighted) 5-9 T2 lesions; ≥10 T2 lesions

##### Gadolinium (Gd)-enhancing lesions, infratentorial lesions, and spinal cord lesions

In a recent study by Brownlee et al. (2019) (53), the presence of Gd-enhancing, spinal cord or infratentorial lesions in relapsing MS patients at diagnosis or early disease (1-3 years) was correlated with poorer long-term outcomes such as conversion to SPMS or increased disability as measured by EDSS. Other MRI studies have also shown correlations between the three lesion types at baseline and disability progression in the first 2-8 years thereafter (52–55), as well as correlations with disability for spinal cord (55–57) and Gd-enhancing lesions (55, 74) alone. Based on a similar premise to the above that these lesion types can be identified using standard MRI protocols, we consider that their incorporation into the prognosis tool offers yet another solid set of useful parameters for neurologists to assess long-term outcomes for their patients.

Scoring: Binary outcome: Absent (better prognosis); Present (poorer prognosis).

##### T1 black holes

T1 hypointense (black holes) lesions are the final MRI measure to be included in the prognosis tool. These lesions have been associated with demyelination, axonal loss, and neurodegeneration, and are therefore considered to be markers of irreversible clinical disability (75–77). A review of published papers carried out by Rocca et al. (2017) (77) highlighted an association between black holes and disability outcomes. To this end, we consider that the presence of black holes is a risk factor for disability progression and therefore of poorer prognosis, thus warranting inclusion of this measure in the prognosis tool. Ideally, brain atrophy and rates of annual brain volume loss will form part of the prognosis tool in the future; however, routine brain atrophy measurements in individuals have not yet become a clinical reality.

Scoring: Presence or absence of black holes. Binary answer: Yes/No.

#### Biomarkers

Responses to this section in the prognosis tool can be viewed in **Table 1** (Section D).

##### Oligoclonal bands in the cerebrospinal fluid

In a recent review of studies that examined the prognostic value of immunoglobulin G (IgG) oligoclonal bands (OCBs) in the cerebrospinal fluid (CSF) of MS patients, Magliozzi and Cross (2020) (78) reported that most of these studies associated the presence of OCBs with poorer prognosis. In contrast, the absence of OCBs is correlated to a more benign disease course. Strong evidence of different clinical outcomes in the absence or presence of OCBs was provided by Dobson et al. (2013) (79), whose meta-analysis of 10 studies showed significantly poorer prognostic outcomes (EDSS milestones) in OCB-positive patients. The presence of immunoglobulin M (IgM) bands in particular has been associated with poorer prognostic outcomes (80–82). Given that OCBs can be routinely measured in the hospital scenario, we have included their measurement in the prognosis tool, particularly with respect to IgM OCBs if possible.

Scoring: IgG OCBs Binary: Absent (better prognosis); Present (poorer prognosis).

IgM OCBs: Even poorer prognosis than IgG OCBs when present.

##### Neurofilament light chain levels in CSF or serum

There is some disagreement in the literature over the exact clinical significance of the presence of neurofilament light chains (NfL) in the CSF or serum given that this biomarker, although a good indicator of neurodegeneration, is not specific to MS. However, the weight of evidence from numerous studies involving MS patients points to a correlation of high NfL levels with disability progression. For example, Disanto et al. (2017) (83) showed that serum NfL levels in MS patients that were higher than the 80^th^ percentile in healthy controls indicated a considerably higher risk of increased EDSS. Likewise, Kuhle et al. (2019) (84) reported that high versus low serum NfL levels were associated with a greater risk of confirmed disability worsening at 2 years. The association with disease progression is more robust when composite measures involving NfL and MRI parameters are used (78, 85–88). As CSF and serum NfL levels are highly correlated (78, 83), prognostic testing with blood samples has become a clinical reality (84) and far less invasive than performing a lumbar puncture. Indeed, a recent study by Benkert et al. (2022) (89) showed that serum NfL percentiles and Z scores (established based on a control cohort with no evidence of CNS disease) may permit the identification of people with MS who are at risk of a poorer prognosis. Given that the clinical significance of NfL is somewhat contentious and techniques to assess their levels are not yet widely implemented in routine clinical practice, we suggest that this will be a biomarker to watch in the future. Scoring: NfL levels <80th percentile in healthy controls (better prognosis); NfL levels >80th percentile in healthy controls (poorer prognosis).

#### Optical coherence tomography and evoked potentials

Responses to this section in the prognosis tool can be viewed in **Table 1** (Section E).

##### Retinal nerve fibre layer (RNFL) properties

Many studies have shown that OCT can be used to obtain valuable prognostic information about the MS patient. Baseline RNFL thinning is indicative of subclinical axonal damage and early neurodegeneration that can be measured by OCT. Oreja-Guevara et al. (2012) (90) showed that thinning of the RNFL is present from the earliest stages of the disease (clinically isolated syndrome), while Martinez-Lapiscina et al. (2016) (91) reported that patients with a peripapillary RNFL of ≤88 μm had double the risk of disability worsening after 1-3 years of follow-up, and a nearly 3-fold increased risk of disability worsening from 3-5 years of follow-up.

Scoring: Binary outcome: Presence or absence of an RNFL thickness ≤88 μm at baseline.

##### Evoked potential characteristics

Electrophysiological studies of MS patients *via* the use of EPs offer a relatively straightforward means to assess long-term prognosis. Leocani et al. (2006) (92) showed that abnormal somatosensory (SSEP), motor (MEP), and global (where the different abnormalities (latency, amplitude, form) of the distinct EPs are added together in one score) EP scores at baseline were highly correlated with disability progression at follow-up [30.5 ± 11.7 months (mean ± std dev)]. These observations provide insight into the prognostic value of performing EP tests at baseline in MS patients. More recently, Hardmeier et al. (2017) (93) described the value of using multimodal EPs (mmEPs) as a prognostic biomarker. Here, a combination of different EP modalities is used to provide a measure of functional alterations across different tracts of the CNS. This is important given the heterogeneity of MS and the fact that some tracts may be more affected than others and not necessarily identified using single EP modalities. To make the prognosis tool as practical as possible, we suggest that neurologists record as many types of EPs as are available to them, including visual EPs (VEPs), SSEPs, MEPs and to a lesser extent brainstem auditory EPs (BAEPs). The number of EPs showing latency, amplitude or morphological abnormalities should be counted, with the presence of 0 or 1 abnormal EP indicative of a better prognosis. In contrast, we feel that two abnormal EPs, and more particularly three or more abnormal EPs would suggest a poorer prognosis as per Pelayo et al. (2010) (94). Keep in mind that MEP and SSEP scores at first presentation were shown to correlate significantly with EDSS values after five years (95) and could therefore be expected to have the highest impact on prognosis.

Scoring: We suggest that ≤1 abnormal EP is associated with a better prognostic outcome, while two or ≥3 abnormal EPs would denote a progressively poorer outcome.

## Discussion

We have presented here a prognosis tool which we believe should enable the MS neurologist to optimally profile newly diagnosed MS patients and to consequently define appropriate treatment strategies relevant to each patient’s disease status. DMT indications tend to be based on patients’ current disease status as evidenced by clinical and imaging findings. However, we contend that ‘disease activity’ at MS diagnosis and ‘poorer prognostic signs’ are not one and the same thing. A patient with a poor prognosis may not necessarily have ‘highly active disease’ at diagnosis, but may exhibit, as we have shown here, numerous other disease characteristics requiring a more proactive treatment approach. Such an approach demands a higher degree of vigilance by the neurologist that is guided by the severity of the prognostic factors defined here in the tool, enabling the MS neurologist to provide a level of care superior to that of simply administering DMTs in order of efficacy as per local prescribing guidelines. A key objective of this strategy is to stop or slow-down disability progression early in the disease in order to prevent transition to SPMS. It is well recognised that most currently available DMTs primarily address the peripherally-driven focal inflammatory component of MS typically seen in relapsing forms of the disease, with the expectation being that disability progression can be minimised if this activity can be controlled. The importance, therefore, of a proactive approach on the part of the neurologist to effectively control early focal inflammatory activity is crucial from the outset.

The inherent value of this prognosis tool lies in the fact that most, if not all of the parameters we have chosen for the tool can be measured using clinical, biochemical, and imaging procedures/techniques that now form part of standard practice in many parts of the world. Implementing these tests in the newly diagnosed MS patient should orientate the MS neurologist to the patient’s likely prognosis (if left untreated or inadequately treated), and to identify ‘red flags’ in the patient’s profile indicating the need for heightened vigilance and/or a more effective treatment approach.

While other authors have addressed in various ways the topic of the prognostic value of individual or grouped clinical and imaging parameters (see e.g (2, 7, 14, 16, 18, 35, 46, 48, 50, 51, 53, 60, 65, 66, 78, 92, 96–100), our aim here was to provide the MS neurologist with a tool in the form of a printable document that can be completed by the neurologist or their support staff. By providing a visually descriptive output, the document should orient the neurologist to the real prognosis for each patient, to treatment approaches that should be considered, and, where appropriate, to explain to patients why one treatment strategy might be a preferred option over another.

Our prognosis tool has some limitations that should be noted. For example, although backed by literature reports and many years of clinical experience on the part of the authors, the tool has not been scientifically validated. Moreover, while the parameters used will orient the neurologist to potentially poor or better prognostic outcomes in specific patients, the presented responses have not been weighted on the basis of their importance to overall prognosis. The validation and weighting aspects of the tool will form the basis of future work to be performed.

In summary, this MS prognosis tool brings together a considerable amount of data specific to each MS patient, thereby providing the MS neurologist with a comprehensive overview of each patient’s current and potential disease status in the future. The tool should also facilitate the development of personalised treatment approaches based on individualised prognostic evidence, enabling outcomes for MS patients to be optimised.

## Author contributions

Development of the tool was led by BW, with input from all other authors at regular meetings. BW wrote the manuscript, with feedback on early versions provided by H-PH and CO-G. All authors contributed to manuscript revision and read and approved the submitted version.

## Funding

Medical writing support for the preparation of this manuscript along with the payment of publishing fees were provided by the ParadigMS Foundation.

## Acknowledgments

The creation and communication of educational materials by the ParadigMS Foundation is sponsored by Sanofi, Roche, and Merck.

## Conflict of interest

The authors declare that the research was conducted in the absence of any commercial or financial relationships that could be construed as a potential conflict of interest.

## Publisher’s note

All claims expressed in this article are solely those of the authors and do not necessarily represent those of their affiliated organizations, or those of the publisher, the editors and the reviewers. Any product that may be evaluated in this article, or claim that may be made by its manufacturer, is not guaranteed or endorsed by the publisher.
